# Viral Tracing Gone Viral: Expanded Possibilities Call for Increased Caution in Describing Cerebellar Network Connections

**DOI:** 10.1007/s12311-026-02089-w

**Published:** 2026-09-29

**Authors:** Tom J. H. Ruigrok

**Affiliations:** Department of Neuroscience, Erasmus MC Rotterdam, Rotterdam, The Netherlands

**Keywords:** Cerebellum, Cerebellar nuclei, Neuroanatomy, Tracing, Adeno-associated virus, Rabies virus, Pseudorabies virus

## Abstract

This review discusses the huge expansion of using cleverly constructed neurotropic viruses and viral transgenes in neuroanatomical research, with a special focus on the cerebellar field. Thanks to the near-limitless possibilities these new tracing techniques offer, vital new information on connections and functions within the brain are rapidly becoming available. However, tracing techniques in general and viral tracing techniques in particular, can harbor some pitfalls and drawbacks that need to be carefully evaluated in order to come to the correct interpretation of obtained results. After providing a general introduction, this review will briefly discuss some of the most commonly used viral tracers with special focus on their individual characteristics and point out some, potentially unexpected, limitations. Finally, we discuss recent observations concerning some newly described cerebellar connections, mostly obtained with viral tracing, that should be treated with caution and need careful replication. We conclude that only after proper evaluation of tracing results, the correct construction of complex anatomical networks and its subsequent understanding will be possible.

## Introduction

In order to fully understand the functioning of the human brain, intimate knowledge is required on how its constituents, i.e. the neurons, communicate with each other. Neuroanatomy is the branch of neuroscience that specifically deals with a description of the connections within the nervous system and can be considered one of the oldest branches within the vast array of modern neuroscientific research. However, although anatomical descriptions of the human body nowadays are exhaustive, the anatomy of the human nervous system still has to deal with many unknowns.

To some neuroscientists this may come as a surprise as they point to many, often excellent, neuroanatomical textbooks that describe the various functional systems and the fiber bundles that interconnect these systems. In this way they provide a lattice for understanding the flow of information within the central nervous system. However, this information was mostly based on animal research obtained in the former century, using techniques that were not perfect as they frequently did not or could not take into account the complex composition of the studied brain areas. These techniques involved microdissection, ablation, degeneration, and bulk anterograde or retrograde tracing using injection of transportable and recognizable substances into specific brain areas. Although synthesis of these results provided a skeleton overview of brain anatomy that served other aspects of the neuroscientific field, it is also clear that more detailed descriptions were required in order to understand the neuroanatomical foundation of their physiological and behavioral impact on animals.

These more detailed descriptions became possible in the new millennium with a surge of new tracing techniques that has provided a wealth of additional new data that, to a large extent, still need to be incorporated in our textbooks. These techniques are based on neurotropic viruses that can replicate within infected neurons, while some of them also can pass between connected neurons. As such, they can be used to visualize and track specific neurons throughout the brain. Thanks to their replication, transneuronal transfer, and genetic engeneering potential, they exhibit increased sensitivity and selectivity, especially when used in genetically modified mice. Indeed, these techniques have truly revolutionized our neuroanatomical knowledge. However, in our view, it also harbors some dangers that we feel need to be addressed. Here, we will provide an overview of some frequently used viral tracers and point out some potential pitfalls that researchers should be aware of when applying these techniques. Furthermore, in light of this, we will discuss some recently described new findings concerning the organization of cerebellar outputs.

## Types of Viral Tracers

Ideally, neurotropic viruses that can be used for neuroanatomical research need to be simple to manage and handle in the laboratory and easy to introduce into confined places of the central nervous system, where it should be able to incorporate itself within neurons. Moreover, either it should be able to multiply itself, preferably without disturbing the function of the neuron itself, or it should be able to introduce a payload that can make use of the cellular production machinery. Ultimately, the virus, or its payload, needs to be visualized, preferably together with other recognizable aspects of the anatomy of the nervous system. Indeed, in many instances, genetically engineered viruses can be turned into viral vectors that are not, or not immediately, pathogenetic and produce their own marker for identification. Presently, most viruses used for tracing are several modified strains of adeno-associated viruses (AAV) [1–4] and strains of rabies virus (RABV) in wild-type (i.e. non-engineered) or genetically engineered form [5–8]. Furthermore, pseudorabies virus (PRV) and several strains of Herpes Simplex virus (HSV) are used as neuroanatomical tracers [9–11]. Although some viral tracers can be used in rats and monkeys [12, 13], most viral tracing studies are executed in wildtype or transgenic mouse lines.

### Comparing Viral Tracers With Classic Ones

In order to assess the quality and usefulness of neuroanatomical tracers it would be vital to understand its uptake and transport mechanisms. Unfortunately, especially the uptake or, perhaps better, the actual process of infection of a neuron in many instances is not clear [14–16]. Importantly, a viral tracing technique has to be qualified as resulting in anterograde, retrograde or bidirectional tracing. In addition, it has to be determined if, and if so to what extent, it is able to infect other neurons transneuronally in an anterograde (i.e. from axon terminal to postsynaptic neuron), retrograde (i.e. from soma or dendrite to presynaptic axon terminal), or in both directions.

A good way to assess the characteristics of viral tracers would be to compare them with the characteristics of well described, so-called, classic tracers like horseradish peroxidase coupled to wheatgerm agglutinin [17] and cholera toxin B subunit [18], both used as bi-directional tracers; Phaseolus vulgaris leucoagglutinin [19] and biotinylated dextran amine [20], both mostly used as anterograde tracers; wheatgerm agglutinin coupled to gold particles [21] and several classes of fluorescently coated latex beads or gold particles [22, 23], all used as retrograde tracers. Further information on properties and the use of classic tracers can be found in many excellent reviews [24, 25]. Unfortunately, only a few studies really compared the labeling characteristics of these classic tracers with those of the viral vectors in a similar experimental paradigm [26–28]. From these studies, it is generally concluded that viral tracing is as good or better than the classic techniques. Nevertheless, discrepancies are also noted and suggest that the results of viral tracing should be evaluated with great care. Some examples will be discussed below.

### Viral Infection of Cell Types

It is important to assess how virus particles enter their host cell and if there are differences in their entry of different types of cells. Also, it may be that the rate of infection shows differences for different types of cells. Some viruses only seem to infect neurons whereas others may also infect all or specific types of glia. In the latter case it would be relevant to assess if glial infection occurs from neuron to glia or vice versa or if the virus can also move from glial cell to other glial cells of the same or other types. E.g. in the case of the Bartha strain of PRV, it was observed that the virus could enter but apparently not replicate within astroglia, thus preventing transneuronal infection through non-synaptic spread [29]. However, a more significant characteristic of PRV might be that its sensitivity for transfection of sensory and autonomic systems seems to be much higher than for motor systems [15, 30]. Indeed, many studies have used PRV specifically to study the innervation of organs and smooth muscles [31–35]. However, it also implies that autonomic innervation of blood vessels within and around those organs may hamper conclusions on the innervation of these PRV-injected structures. In addition, as PRV does not infect neurons in primates, it cannot be used to study the primate nervous system. Finally, different strains of PRV have different characteristics in speed and direction of transmission, all of which should be taken into account for proper evaluation of the experimental results [13, 15].

Similar restrictions are also seen in the use of various strains of HSV, which are detailed in a comprehensive review by Ugolini [15]. It is striking to conclude that large differences exist in infection proficiency, direction of transport, ease of either anterograde, retrograde or indiscriminate transneuronal infection and spread of virus by way of infection of glial cells. Moreover, in most instances, cellular infection by HSV strains, like that of PRV strains, usually results in rapid degeneration of the infected cells, enabling uptake of viral particles by surrounding neurons [13, 36].

**Fig. 1:**
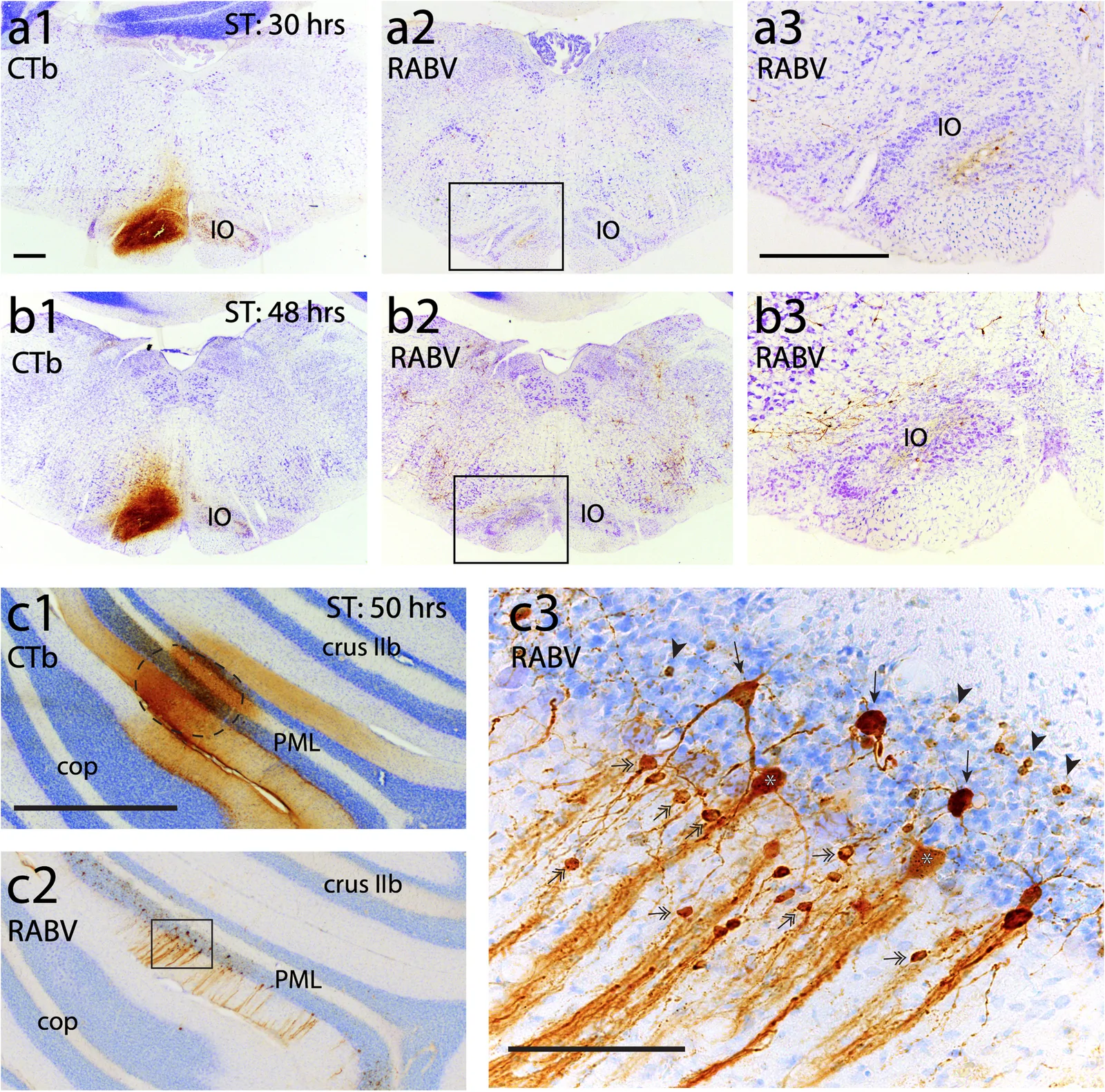
The effective injection site of RABV is difficult to establish. **a1-3**: injection site 30 h after injection of 150 nl of a mixture of RABV and CTb into the inferior olivary complex (IO) of the rat. **a1** shows the injection site in a section incubated with anti-CTb, **a2** shows an adjoining section of the same animal reacted with anti-RABV. Boxed area is enhanced in **a3**. Note that only a few RABV-positive neurons can be appreciated located between the medial accessory and principal olives. **b1-3**: Similar in another case with a survival time of 48 h, sufficient for a second retrograde transneuronal infection step. Note the abundance of RABV infection in the reticular formation and just outside the IO. However, still virtually no IO neurons were RABV-positive (**b3**). **c1-c3**: Similar after an injection of 150 nl RABV/CTb mixture in the paramedian lobule of the rat. CTb immunohistochemistry shown in **c1** allows the delineation of a circumscribed effective injection site (hatched line). **c2**: RABV immunohistochemistry of an adjoining section shows a wide area of infected neurons without allowing a description of the effective injection site. **c3**: Note that several cell types are RABV-positive, such as Purkinje cells (asterisks), Golgi cells (arrows), granule cells (arrowheads) and molecular layer interneurons (double-headed arrows). For further information see Ruigrok et al. [46]. Scale bars in a1, a3 and c1 equal 500 μm, and 100 μm in c3. Abbreviations: cop, copula; PML, paramedian lobule. Adapted, modified and relabeled from Ruigrok et al. [46]

Retrograde transneuronal transport of RABV has been reported to be superior to that of the alpha-Herpes Simplex viruses [13, 15]. Important specific characteristics of RABV were its apparent indiscriminate uptake by all cell types (apart from peripheral sensory and autonomic neurons) and its time-sensitive retrograde spread throughout linked connections within the central nervous system [13, 15]. In addition, neurons were reported to survive infection for weeks during which the virus did not leak out of infected cells to surrounding neuronal or glial structures. As RABV had not been reported to fail to label known pathways [13], it would render RABV a highly attractive instrument to study linked connections in the nervous system. An obvious drawback of working with RABV are its strict handling requirements necessitating specific safety precautions and laboratory use [12]. Yet, under the required circumstances, using various laboratory strains of unmodified RABV, retrogradely connected networks have been described starting from injections in muscles [37–40], but also from injections in specific brain areas [41–45]. From these studies, it appears that the virus gains access to the neuron by entering the terminal boutons, presumably using the neuronal cell adhesion molecule NCAM as a mediator [14, 15]. Yet, in the central nervous system, infection of somata and dendrites at the injection site, however, is usually very poor, making it difficult to establish the effective site from which uptake by axon terminals has occurred [14, 15] (Fig. 1). In addition, it has become clear that different strains of RABV, or even the use of the same strain in different species, may produce different results.

**Fig. 2:**
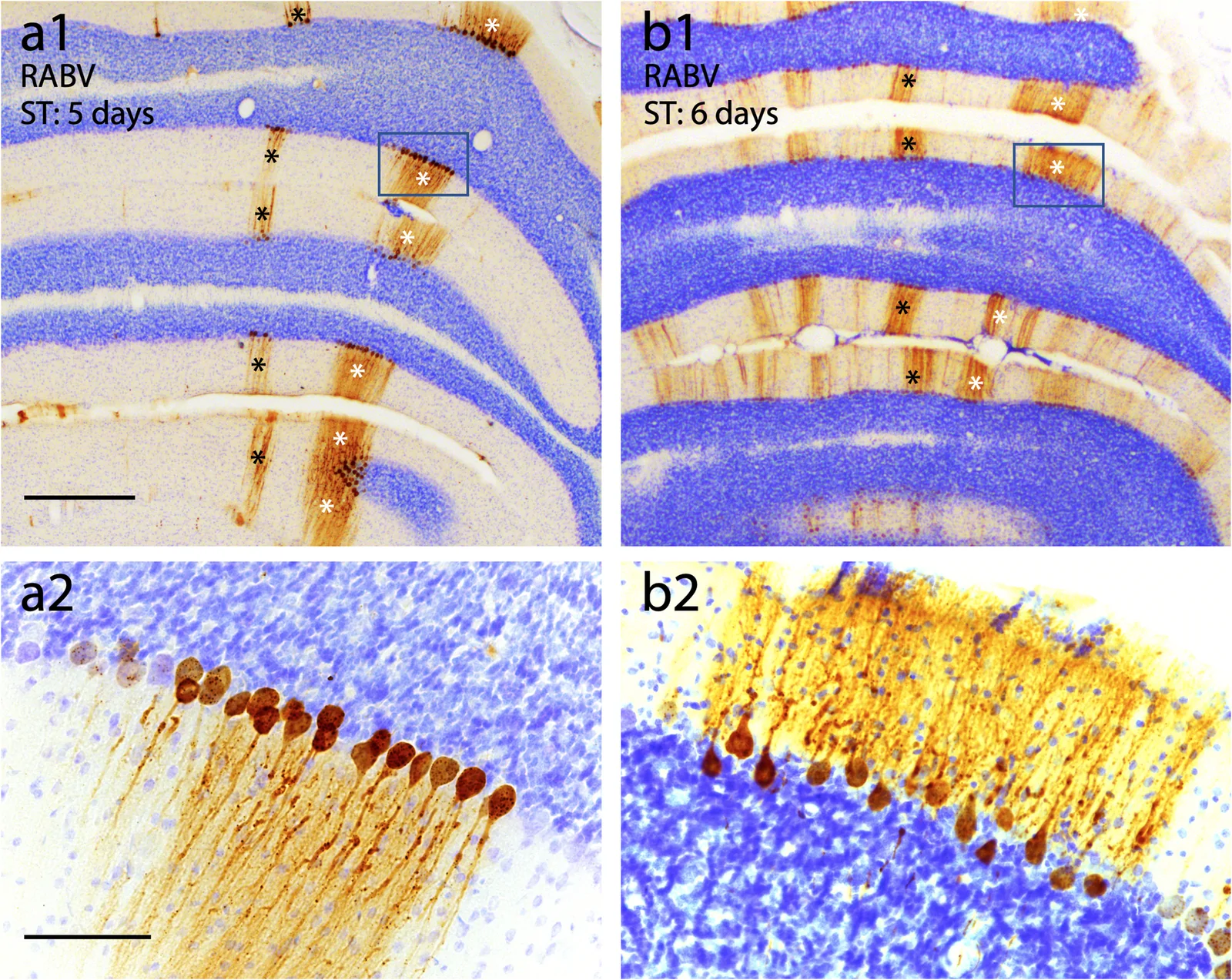
Failure of some cell types to become infected by RABV. **a1**: First appearance of infected Purkinje cells after RABV injection into the tibialis anterior muscle of the rat 5 days previously. Black asterisks indicate the lateral A1-zone, whereas the strip indicated by white asterisks could be identified as the B-zone. Boxed area is magnified in **a1**. **b1**: Six days after injection many more strips Purkinje cells have become RABV-positive, indicative of a further transneuronal step beyond the first labeled Purkinje cells of the lateral A1- and B-zones. However, no neurons presynaptic to the Purkinje cells of these zones, such as granule cell or molecular layer interneurons have become infected by RABV as can be appreciated by the lack of RABV infection of other than Purkinje cells in **b2**. This indicates that the chain of time-locked transneuronal infections has become ineffective. Scale bars in **a1** and **a2** equal 500 and 100 μm, respectively. Adapted, modified and relabeled from Ruigrok et al. [46]

As an example, we note that cerebellar granule cells, which are presynaptic to Purkinje cells, can be infected with the Challenge Virus Standard (CVS)−11 strain of RABV after injections in the cerebral cortex as fourth-order labeling in monkey [42], and were also reported 3–5 days after CVS-11 RABV injection of respiratory muscles in the adult mouse [47]. However, no granule cells were observed in rat in instances where infection of Purkinje cells, resulting from injections of CVS-11 in limb muscles, was noted 40 to 72 h earlier [40, 46]. Likewise, no RABV-infected molecular layer interneurons (i.e. stellate and basket cells), known to be synaptically linked to the previously infected Purkinje cells, were observed in these cases (Fig. 2 b2). Yet, RABV injections directly into the cerebellar cortex, readily resulted in infection of these cell types (Fig. 1 c3). This suggests that species differences may affect the propensity of this strain of RABV virus to transfect connected neurons from a particular cell type (in this case Purkinje cells).

**Fig. 3:**
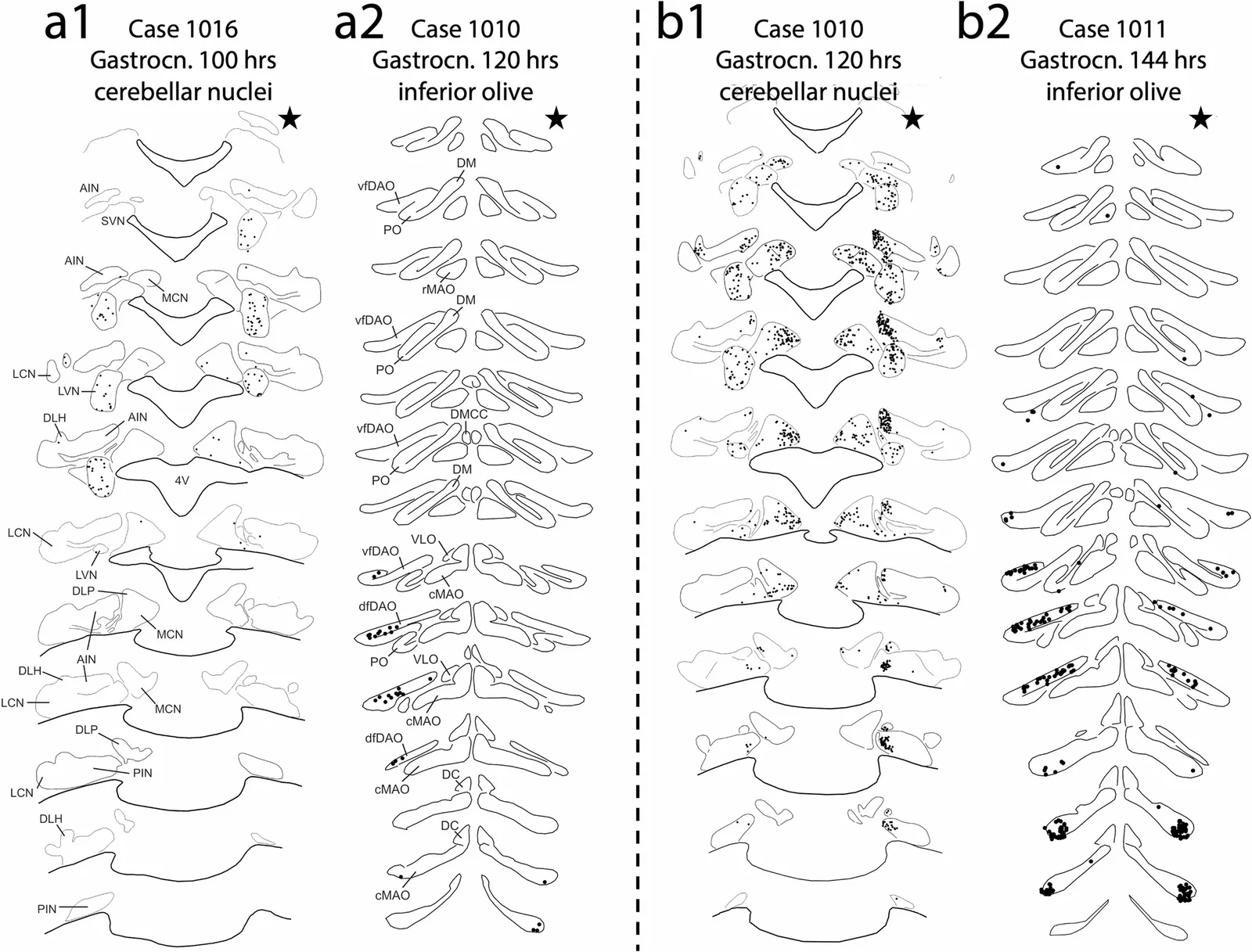
Inferior olivary neurons in the rat can show resilience against RABV infection. Four series of plots are from three different cases with a RABV injection of the gastrocnemius muscle but each with a different survival time. **a1**: Pattern of RABV-infected neurons in the cerebellar nuclei and LVN after a survival time of 100 h (case 1016). Note that infection was mostly observed within the LVN. At this time no infection was observed within the inferior olivary complex (not shown). **a2**: Another experiment (case 1010) allowed a survival time of 120 h, thus enabling an extra transneuronal passage which, indeed, resulted in infection of IO neurons located within the dfDAO. This matches with the observation that neurons of the dfDAO possess collaterals that terminate within the LVN [49]. **b1**: In the same animal, thanks to the extra transneuronal transfer of the virus many more areas in the cerebellar nuclei, in particular within the interposed nuclei, an abundance of infected neurons was observed. **b2**: However, increasing the survival time still an extra 24 h (case 1013), thus allowing for an additional transneuronal transfer, hardly resulted in infection of neurons in other olivary divisions. In particular the lateral part of the vfDAO remained devoid of infected neurons, despite its collateralization to infected neurons within the medial part of the anterior interposed nucleus shown in **b1**. Also, despite infection of neurons in the posterior interposed nucleus (see **b1**), no corresponding labeling in the rostral part of the medial accessory olive was noted 24 h later [49]. Yet, in the cerebellar cortex Purkinje cells were infected that were related to the the infected areas in both interposed nuclei [40], thereby showing that the survival time of 144 h really was sufficient for a further transneuronal step. This suggests that, although olivary neurons apparently can become infected, they may show resilience against infection. Adapted and modified after Ruigrok et al. [40]. Star indicates injected side. Each dot represents one infected neuron. Abbreviations: 4 V, 4th ventricle; cMAO, caudal part of medial accessory olive; DC, dorsal cap; dfDAO, dorsal fold of the dorsal accessory olive; DLH, dorsolateral hump; DLP, dorsolateral protuberance; DM, dorsomedial group; DMCC, dorsomedial cell column; LCN, lateral cerebellar nucleus; LVN, lateral vestibular nucleus; MCN, medial cerebellar nucleus; PIN, posterior interposed nucleus; PO, principal olive; rMAO, rostral part of medial accessory olive; SVN, superior vestibular nucleus; vfDAO, ventral fold of the dorsal accessory olive; VLO, ventrolateral outgrowth

The sensitivity for infection can also differ between cell types. As Purkinje cells readily became infected from RABV-infected neurons in the cerebellar nuclei, neurons in the inferior olive, known to have connections to both neurons in the cerebellar nuclei and to Purkinje cells [48], were only infected sporadically (Fig. 3). However, olivary infection from CVS-11 RABV injections directly made in the cerebellar cortex occurred much more readily [45, 46]. This suggests that access of this particular strain of RABV to the terminals seems hampered from the RABV infected neurons to their presynaptic olivary terminals, whereas the virus is better able to infect olivary neurons from the extracellular environment of their terminals.

**Fig. 4:**
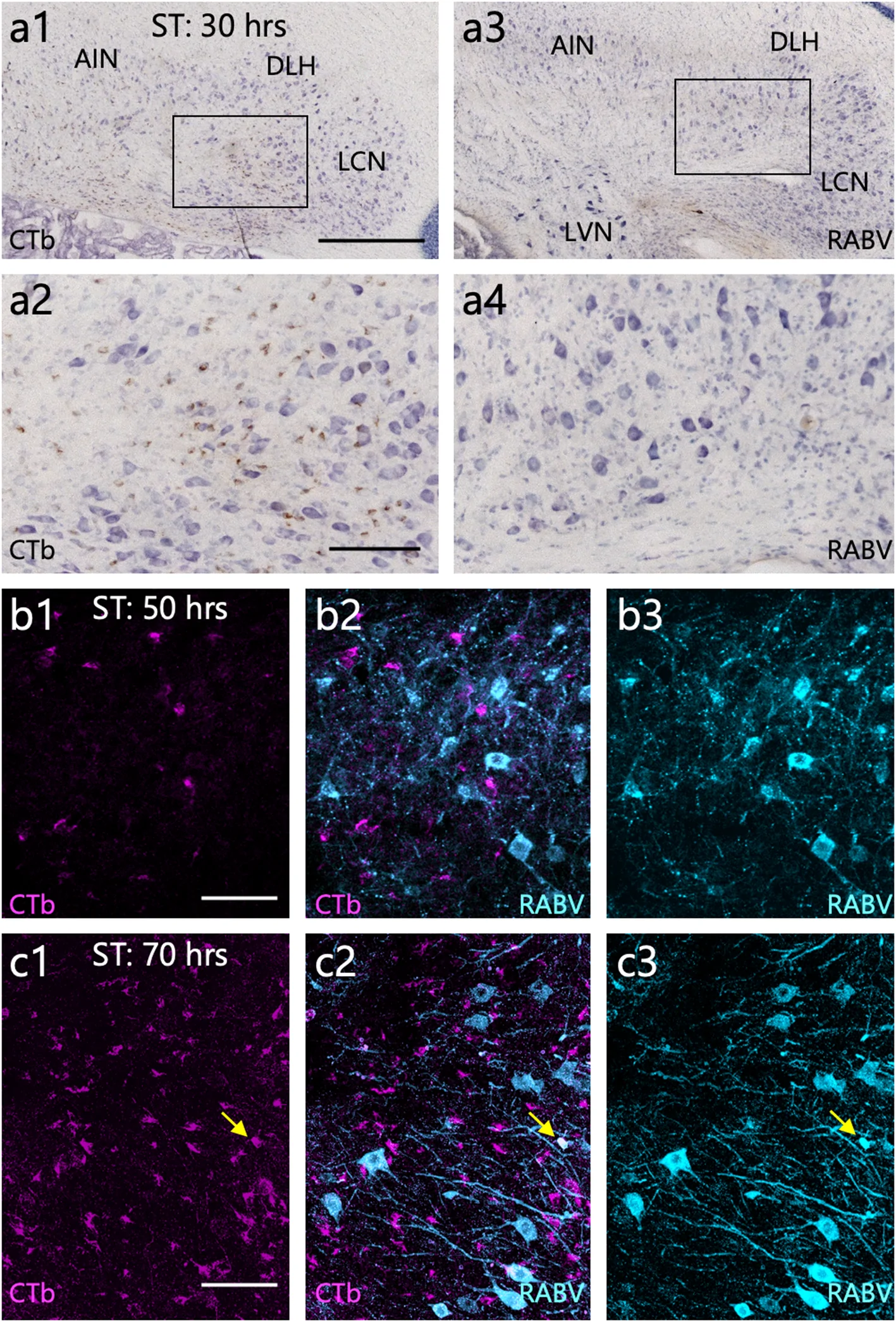
Olivary projecting neurons in the cerebellar nuclei resist infection by RABV. **a1-a2**: overview and detail of part of the cerebellar nuclei after injection of 150 nl of a mixture of CTb and RABV into the inferior olivary complex (CTb and RABV appearances of the injection site are shown in Fig. 1** a1 and 1a2**). Note that intermingled throughout large parts of the cerebellar nuclei small CTb-positive neurons can be observed, which are typical of the small GABAergic neurons that innervate large parts of the inferior olive [51, 53]. **a3-a4**: Overview and detail of an adjacent section incubated for RABV-immunohistochemistry. Note that in a similar part of the nuclei, no RABV-infected neurons are observed. In this animal RABV infected neurons were observed in other areas known to project to the inferior olive [46]. **b1-b3**: Allowing for an extra transneuronal step by increasing the survival time (ST) to 50 h, did result in large RABV-infected neurons within the cerebellar nuclei (**b3**) as well as small CTb-labeled neurons (**b1**) as shown in this double incubated section. However, no double labeled neurons were observed (**b2**), suggesting that the monosynaptic nucleo-olivary neurons infected labeled with CTb are still RABV-negative. The large-sized RABV-infected neurons are suggested to represent projection neurons that targeted RABV-infected olivary afferents located in the midbrain and thus represent second-order transneuronal infection. **c1-c3**: Similar to **b1-3** but now allowing for another transneuronal step (ST: 70 h). Note that again no significant number of double labeled neurons were observed. In this view only a single small double labeled neuron (arrow) was seen. Despite the fact that the small neurons readily can take up CTb at the injection site, these cells apparently resist infection by RABV. Scale bars in **a1**, **a2**, and **b1** and **c1** equal 500, 100 and 50 μm, respectively. Abbreviations as in Fig. 3. Adapted and modified after Ruigrok et al. [46]

In other instances, however, access of the virus to presynaptic neurons seems to be specifically hindered from the extracellular matrix as was observed after injection of the CVS11 strain of RABV into the inferior olive of the rat. Combining injections of RABV with CTb resulted in ample retrograde labeling of a population of small-sized neurons in the cerebellar nuclei with CTb. These cells are known to represent GABAergic neurons that specifically target the inferior olive [50–52]. However, RABV immunohistochemistry 30 h after injection (sufficient for infection of other monosynaptically connected afferents of the inferior olive), 50 h or even after 70 h (sufficient for disynaptic and trisynaptic RABV infection, respectively) failed to demonstrate a population of small-sized RABV-infected neurons in the cerebellar nuclei (Fig. 4) [46]. This suggests that the virus particles were unable or severely restricted in entering the axon terminals of the nucleo-olivary neurons. As at the injection site only a few olivary neurons were found to be infected, it does not necessarily imply that a retrograde transsynaptic passage of the virus from the olivary neuron to the presynaptic nucleo-olivary projecting cell was not at all possible. It was suggested that, as the nucleo-olivary terminations are mostly found within specific glomerular synaptic structures that are enwrapped in a glial sheath within the olivary neuropil [50], this could prevent viral access to the nucleo-olivary terminal boutons [46].

Here, it should be noted that, apart from the CVS-11 strain, which, as detailed above, shows differences in uptake and transfection capabilities between animal species as well as between cells types, there are several other so-called ‘fixed’ strains of RABV (such as the N2c and B2c strains of CVS-24), which, although they are ‘fixed’ by repeated passage through specifically selected media and therefore are supposed to have uniform characteristics, still may display different transfection characteristics [12, 15]. These differences may be enlarged between different viral strains or, potentially, when modifying virus by genetic engineering. As many applications of the G-protein deleted strains of RABV are based on the attenuated Street Alabama Dufferin (SAD) B19 vaccine strain [8], one cannot help but wondering to what extent modified versions of this strain, upon the in-cell recombination with the required G-protein, are able to transfect their presynaptic terminals without discrimination. Indeed, infection using modified versions of SAD B19 has been reported to be sparse and slow [7]. Moreover, infected neurons show early signs of degeneration [8, 54]. In contrast, using G-deleted virions based on the N2c strain of CVS-24 both considerably increased retrograde transneuronal transfer and the viability of the infected neurons [55]. However, no information seems to be available if this, or the SAD B19 G-deleted strain of RABV, possess indiscriminate transfection potential in different brain areas or cell types, and/or in different species.

In this respect a warning seems to be warranted that the use of nearly unlimited genetic engineering of neurotropic viruses should be accompanied by strenuous controls to assess if, or to what extent, their uptake and transport characteristics are still in line with the expectations the authors harbor, especially when also using these viruses in genetically engineered mice. As an example we refer to the influential but recently debated observation that noradrenergic neurons in the locus coeruleus receive a sizable direct input from Purkinje cells. This connection has been suggested using new and fairly complex viral tracing techniques in dopamine β-hydroxylase-Cre (Dbh-Cre) transgenic mice [56, 57], but this suggestion was severely and rather convincingly downplayed in a recent publication using different techniques [58].

### Adeno-associated Viruses

That different viral strains may have widely differing tracing characteristics is also nicely illustrated by the various serotypes of recombinant adeno-associated viruses (AAV) that are used. As AAV’s can infect, but do not replicate within neurons, they are mostly used to deliver transgenes coding for fluorescent proteins to the host cell, which will subsequently be expressed. As such, usually after a period of several weeks, sufficient expression ensures the visualization of the infected cell body, dendrites and complete axon and its terminal arborizations. However, different AAV serotypes vary in their propensity to infect specific cell types. These differences in tracing characteristics are thought to depend on modifications of viral surface capsids that respond to specific surface proteins of the neurons [16] and their use for neuroanatomical tracing seems to be mostly empirically established. As an example, it is not clear why a strain of AAV1 expressing EGFP, used as an anterograde tracer to establish the Mouse Connectivity Atlas and accessible at the online database provided by the Allen Brain Institute [26], does not visualize any postsynaptic neurons, whereas another strain of the AAV1 serotype, expressing Cre, succeeds in the postsynaptic expression of the Cre-dependent tdTomato reporter in Ai14 mice [4].

Whereas the CVS11 strain of RABV does not, or only poorly, infect neurons within the injection site, injected AAV1 infects local somata and/or dendrites relatively easily, upon which the cell starts to produce the fluorescent proteins in great quantities which, subsequently make use of the anterograde transport mechanism to label axons and terminal arborizations. As such, the injection site can clearly be demarcated by the dense cluster of AAV1 infected neurons. However, especially in the light of the relatively long survival times, one wonders how long and to what extent the infectious AAV particles may travel within the brain’s extracellular space and reach nearby other brain regions in order to infect neurons. It will be obvious that infected neurons outside of the densely labeled injection site also may result in definite and noticeable anterograde labeling that may be quite different from that coming from the center of the injection site. In this respect, the evaluation of the effective injection site of viral tracers may be different from that of more classical tracers like BDA, Pha-L or CTb, as visualization of these tracers within labeled neuronal structures will, apart from the sensitivity of the used detection technique, be critically dependent on the concentration of incorporated tracer, which will considerably fall at the edges of the injection site, resulting in less conspicuous or ineffective uptake.

Other AAV serotypes, like AAV2, 5, 8 and 9, are reported less likely to infect neurons as determined by their expression of reporter genes [3] or to display a preferential neurotropism for specific cell types [59]. Indeed, as discussed above for RABV, differences in infection effectiveness may exist in the size of the apparent injection sites and results may differ between different brain areas, cell types and anterograde and retrograde transport characteristics [3, 27, 60]. Importantly, although the neurotropic effectiveness as an anterograde tracer between various strains differed, all versions also showed some propensity of retrograde labeling, i.e. initiated by infection via axon terminals and propagation to the soma to induce gene expression [27].

Highly improved retrograde tracing has been reported in a recombinant AAV variant typed AAV2-retro strain [61]. After injection, AAV2-retro can infect neurons by specifically gaining access to their axon terminals, and travel retrogradely to its soma for expression of their payload. Yet, in a study comparing the effectiveness of retrograde tracing by AAV2-retro, RABV-* G* and fluorescent latex beads, similarities but also labeling differences were noted in several areas, suggesting that each established retrograde tracer still has its own preferences and characteristics [27].

It should be noted that the effective injection site of viral tracers that can only infect neurons by their axon terminals will be notoriously difficult to establish (as already described for wildtype RABV, see Fig. 1). Usually, another (viral) tracer, that can infect neurons by entry of their dendrites or somata or substances that do not easily degrade (like fluorescent beads) are co-injected in order to visualize the injection site. Yet, different viruses or substances may have different diffusing characteristics or take different times to degrade resulting in different spread of the injectate [15, 62].

### Sensitivity and Selectivity of Labeling By Viral Tracers

A major advantage of viral labeling either using antibodies against viral proteins or by transgenic inclusion of reporter genes is its self-multiplying capacity or by unbounded expression of the reporter gene. After transport and distribution throughout the entire infected neuron this may result in easy visualization of even the smallest branches of dendritic tree or terminal arborizations, including collaterals. As such, the labeling may stand out more profoundly than that of tracing by non-multiplying classic tracers, such as WGA-HRP, CTb or BDA. In addition, using Cre-dependent reporters and transgenic mice, viral tracing now is possible in selected, type-dependent groups of neurons. The enhanced sensitivity of tracing specific groups of neurons enabled the recent surge in describing many more, and more complex, connections.

A prime example is the recent discovery that some neurons in the cerebellar nuclei of the mouse have axons that descend and terminate as far as the lumbosacral spinal cord [63], projections that have not been described before in other species [48, 64, 65]. Similarly, now by using various viral transgenes and mutant mice, it was recently shown that some GABAergic neurons that were thought to only project to the inferior olive [53, 66], possess collaterals that terminate in other brainstem areas such as the red nucleus and spinal trigeminal nucleus [67]. Likewise, it has appeared that not all projections from the cerebellar nuclei to the inferior olive originate from GABAergic neurons [68].

However, as outlined above, the use of viral tracers, though enhancing both sensitivity and enabling increased selectivity to the observed tracing, also may harbor some dangers that need proper evaluation and may require additional controls before coming to firm conclusions. Ideally, unexpected findings should be replicated by other techniques and investigators. Furthermore, they should be confirmed in other animal models and supported by human studies where feasible. Below, we discuss three examples of recently described connections from the cerebellar nuclei to areas that were previously not considered to be typical output stations of the cerebellar nuclei.

#### Is There a Major Direct Projection From the Cerebellar Nuclei to the VTA and SNc?

As a first example, we have noted that recent publications of a major cerebellar nuclear projections to the ventral tegmental area [69] and substantia nigra pars compacta [70] have received considerable attention [71–75]. Although we do not doubt that cerebellar influence on motivational aspects of behavior will most certainly exist, we have reservations to what extent the cerebellar nuclei have a direct line to VTA and SNc. Studies employing degeneration or classic anterograde tracers have not convincingly documented a robust projection from the cerebellar nuclei to the VTA in rodents [76, 77], although an anterograde degeneration study in cat and rat by Snider et al. [78] does mention some fine degenerating fibers and terminals in small areas of the VTA and the SNc of the cat. Of course, the superior sensitivity of the viral tracing enabling visualization of even the faintest and smallest fibers or axonal terminations, could have escaped detection by these earlier studies. Obviously, however, sparse labeling of only a few fibers in an area may also put questions on the importance of the observed connection, whereas the connection from the cerebellar nuclei to the VTA has been classified as ‘robust’ [69]. So, how to explain the apparent mismatch between the modern and earlier descriptions.

Here, it should be mentioned that in mice, many studies using fluorescent tracers are making use of automated tracking of labeling by measuring the fluorescence intensity in brain areas that can subsequently be matched to digitized atlas images available online (e.g. from the Allen Brain Institute). It will be obvious that the matching procedure of the actual samples with the digitized images needs to be carefully handled and checked in order to match observed labeling within an identified region. Moreover, these processes usually do not take into consideration that labeling of axonal fibers does not necessarily mean that there are terminal arborizations as determined by the presence of labeled varicosities or synaptic clefts [77]. Hence, passing fibers or slight mismatch between actual samples and atlas may result in long lists of alleged projection areas [79]. Yet, in case of the description of the cerebellar projections to the VTA by Carta et al. [69], labeled terminals within the confines of the VTA were definitely established.

**Fig. 5:**
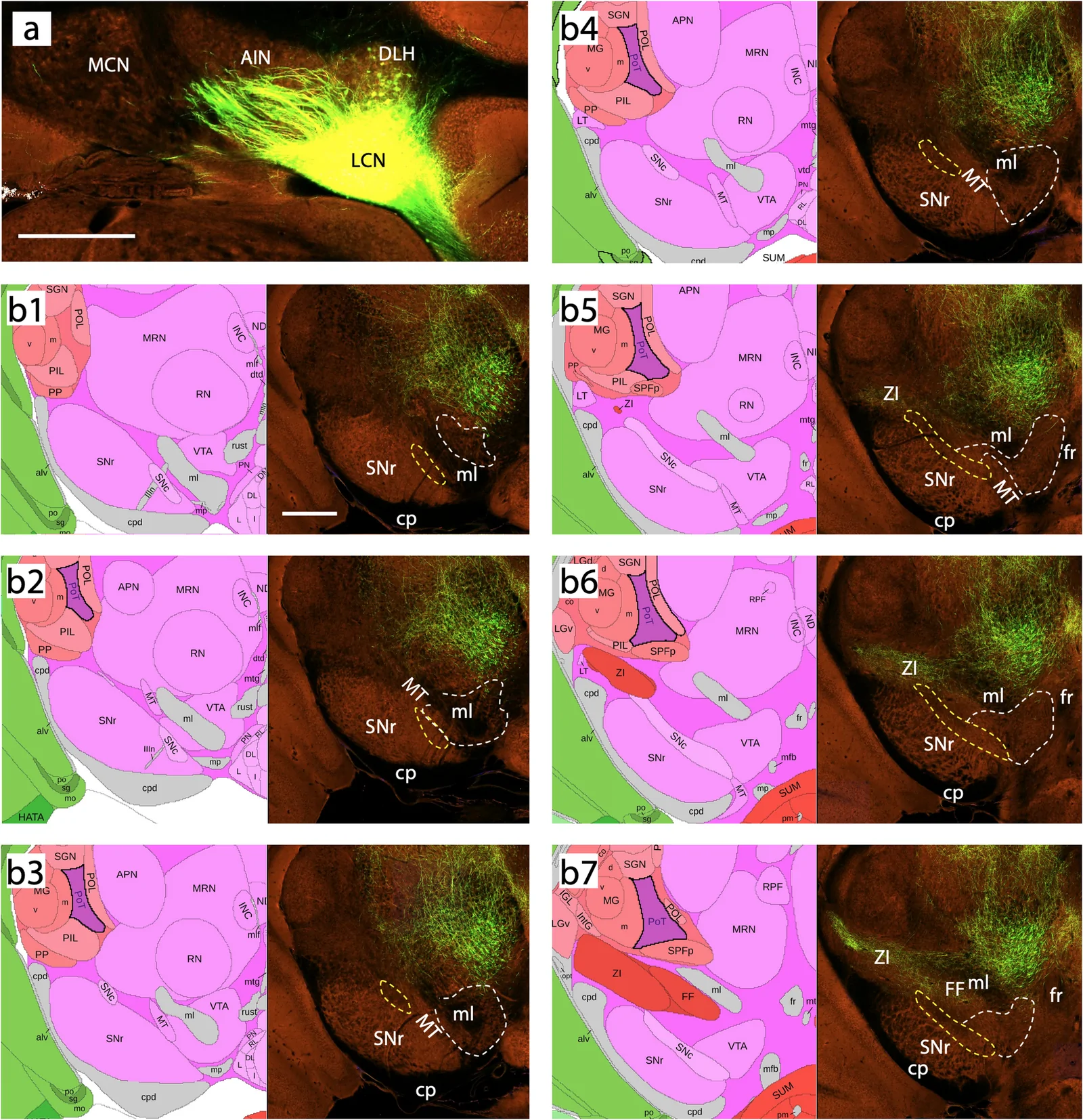
The lateral cerebellar nucleus does not provide a significant projection to the ventral tegmental area (VTA) and substantia nigra, pars compacta (SNc). Material from experiment 268,389,532 , made available by the Allen Brain Institute in the Mouse Brain Connectivity Atlas [26]. **a**: Injection site of 160 nl of anterograde transported AAV-EGFP centered on the LCN of the mouse. In this experiment the parabrachial nuclei were clearly not involved in the injection site (not shown). **b1-b7**: Consecutive sections based on serial two-photon tomography together with the matching atlas representation. The atlas representation was used together with aspects of the tomography pictures to indicate several fiber tracts and nuclei. The outlines of the VTA (white hatched line) and SNc (yellow hatched line) are indicated. Note the abundance of labeling directly dorsal to the outlined areas. An occasional labeled fiber can be seen passing through the outlined areas. However, dense terminal arborizations cannot be observed anywhere within the VTA or SNc. Abbreviations: AIN, anterior interposed nucleus; cp., cerebral peduncle; DLH, dorsolateral hump; FF, fields of Forel; fr, fasciculus retroflexus; LCN, lateral cerebellar nucleus; MCN, medial cerebellar nucleus; ml, medial lemniscus; MT, medial terminal nucleus; SNr, substantia nigra, pars reticulata; ZI, zona incerta. Other atlas abbreviations can be found at the mouse atlas of the Allen Brain Institute. Scale bars represent 500 μm

It is clear that when assessing labeling in any area a concise evaluation of the effective injection site would be of the utmost importance in the case of any used tracer, but, as discussed above, especially when using visualization based on the use of viral transgenes as any infected neuron may result in strong labeling of its axon. In the study by Carta et al. [69] that established a connection between the mouse cerebellar nuclei and the VTA, 300 nanoliter (nl) of the transneuronally anterogradely transported viral tracer HSV129-GFP was injected at two sites centered on the cerebellar nuclei of the mouse (AP −6.0, L 2.3, D, 2.3 and AP −6.0, L 1.2, D 2.1). Although no actual injection sites were shown, it should be noted that 300 nl represents the volume of a sphere with a diameter of more than 800 micrometer. In the mouse, the caudal aspect of the parabrachial nuclei lies approximately 300 micrometer rostral to the center of the atlas-based injected site [80]. As the parabrachial nuclei project abundantly to the VTA bilaterally [81–83], it would be important to rule out the possibility that their terminals might have been involved in the observed results of Carta et al. Especially as the authors injected even larger volumes (up to 2.4 µl) of viral transgenes in their subsequent in vitro electrophysiological and behavioral experiments using optogenetic stimulation. Moreover, the density of channelrhodopsin labeling shown in the supplemental material to the paper seems to be far higher than could be expected based on earlier studies on cerebellar nuclear afferents in rodents [76, 77] or can be determined from the material available online in the Mouse Connectivity Atlas of the Allen Brain Institute [26] (Fig. 5a). Also, a study by Kebschull et al. [79] employing AAV1 injections into the mouse cerebellar nuclei, which, although it might also have been hampered by their 500 nl injection volumes, did not list the VTA within the 40 regions with the highest innervation densities as based on fluorescence intensity. These notions suggest that VTA could hardly qualify as a prominent or ‘robust’ termination area of cerebellar nuclear efferents.

The behavioral studies performed in the study by Carta et al. [69] make use of optogenetic stimulation (excitation and inhibition) by placing the laser fiber optic near the VTA after injection of viral transgenes in the cerebellar nuclei. However, the cerebellar nuclei also send projections to nearby (i.e. within 2 mm) areas of the VTA, such as the periaqueductal gray, superior colliculus, lateral hypothalamic area and zona incerta [77, 84] (also see Fig. 5a). As these areas are also known to supply efferents to the VTA [85, 86], it would have been advisable to check if these areas are not affected by the used stimulation strengths of the laser placed near the VTA and, in this way, could have influenced activity of this region.

Indeed, projections from the cerebellar nuclei to the VTA have been suggested by other studies employing retrograde tracing using classic [78], fluorogold: [85, 87, 88] or viral tracing [71, 81, 89] and were usually noted to originate from the dentate (or lateral cerebellar) nucleus. In rat, using Pha-L anterograde tracing, terminals from the dentate nucleus are prominently encountered just dorsomedial and ventromedial to the red nucleus, at a position directly dorsal to the VTA, in areas known as the medial accessory oculomotor nucleus (MA3) and visual tegmental relay zone (VTRZ), respectively [53, 66]. These areas, like the VTA, also both contain some tyrosine hydroxylase-positive and GABAergic neurons (source: Allen Brain Atlas, exps. 103073 and 071018 02 B07, resp.), which might have been involved in spurious uptake in respective cre-mouse lines. Moreover, the superior cerebellar peduncle, carrying the major contingent of cerebellar nuclear efferent fibers, passes in a wide array just dorsal to the VTA around and through the magnocellular part of the red nucleus [71] and, therefore, could easily have been damaged by penetrating tips of injection devices (Fig. 5). In addition, the area may contain rostral-most aspects of the crossed descending limb of the superior cerebellar peduncle containing cerebellar fibers directed to the pontine nuclei and inferior olive [48, 90]. Hence, a cautious interpretation of all retrograde tracing results seems to be warranted.

Similarly, projections from the cerebellar nuclei to the SNc as recently reported by Washburn et al. [70] should be treated with caution. Again, relatively large volumes of viral tracers (0.4–1.2 µl) were injected in the cerebellar nuclei, which, as outlined above, may have easily involved infection of neurons in the parabrachial nuclei acting as a major afferent source of the SNc [81, 85, 91]. On the other hand, the retrograde viral tracing experiments employing a retroAAV virus injected in the vicinity of the SNc, resulted in such high numbers of retrogradely labeled neurons throughout most of the cerebellar nuclei (cf., their Fig. 4), that it would seem near impossible that anterograde tracing experiments with classic tracers should have missed these terminations in the SNc. Therefore, it would seem possible that spread of the injected tracer to overlying areas of the SNc, such as the retrorubral field, zona incerta or fields of Forel might have been involved and resulted in the abundance of retrogradely labeled neurons in the cerebellar nuclei. Again, as mentioned above, the course of fibers of the superior cerebellar peduncle should also be taken into account. Finally, although the experiments employing reconstitution of G-deleted RABV in dopaminergic cells of SNc seem to clinch the case of a major direct cerebellar nuclear connection to the SNc [70], it was noted that a careful check showed hardly any dopaminergic neurons outside of the SNc to serve as starter cells. However, in this paradigm the number of RABV-infected neurons in the cerebellar nuclei appears drastically reduced and only a few are shown. It seems possible that such a reduced number might have become infected by the few starter cells that were found outside of the SNc.

Hence, although we contend that the functional description of a robust monosynaptic pathway linking the cerebellar nuclei with neurons of the VTA and SNc is highly interesting, we think that these results should be taken with some caution and would benefit from replication in other animal models and/or by other teams. Indeed, it will be exciting to see to what extent the cerebellum impacts reward systems governed by midbrain dopaminergic neurons directly or by way of di- or polysynaptic pathways with these neurons [86].

#### Is There a Projection From the Cerebellar Nuclei to the Reticular Thalamic Nucleus?

As another example warranting carefull evaluation of results obtained with viral tracers, we mention the recent study by Pisano et al., [92] that payed some attention to cerebellar projections to the reticular thalamic nucleus (RTN). This narrow, dorsoventrally elongated nucleus laterally envelopes the dorsal thalamus and consists of GABAergic neurons that project to the thalamic nuclei and, as such, plays a role in curbing the transmission of thalamic information to the cerebral cortex [72]. It is well-known that the cerebellum can influence thalamic processing by its direct excitatory projections to a score of thalamic nuclei [77, 93, 94]. However, here, as well as in other studies, projections to the RTN were not mentioned as part of these cerebellar nucleo-thalamic projections in either rat [76, 77, 95, 96], cat [97, 98] or monkey [99–101]. Yet, apart from the study by Pisano et al., only in a few instances a report of cerebellar projections to the RTN have been made [102–104]. Chan-Palay studied the efferents of the dentate nucleus of the rat using the anterograde tracer ^35^S-methionine. This tracer is visualized by dipping mounted sections into a photographic emulsion after which the sections are stored in the dark for several weeks to allow silver precipitation at the location of the tracer. By the nature of the visualisation technique individual fibers can seldom be precisely localized and usually only an area of labeling can be identified. Conclusive tracing of the ^35^S-methionine-labeled fibers and terminals to the relatively thin stretch of nuclear tissue of the RTN could reflect labeling in the lateral-most aspect of the ventral lateral and ventral anterior nuclei of the thalamus. The study by Cavdar et al. [102] made use of pressure injections of the retrograde tracer HRP injected into the rostral RTN and report labeling of neurons in the interposed and lateral nuclei of the ipsilateral cerebellar halve. It is obvious that dorsal injections into the narrow RTN run risk to encroach upon the medially adjacent thalamic nuclei as can also be surmised from their Fig. 1. Furthermore, the observation that labeled cerebellar neurons were only reported ipsilateral to the injection site is interesting but is at odds with the other claims of a nuclear projections to the RTN, which all specify a predominant contralateral projection [92, 103, 104]. The study by Nakamura [104] was carried out in the cat and specified projections to the ventral lateral geniculate nucleus (LGNv) and RTN. The described projection pattern represented a more or less continuous sweep of fibers and terminals over part of the zona incerta, caudal part of the RTN and LGNv and appears to be located much more caudal than the main body of the RTN as seen in rodents and as claimed to receive nuclear terminals by Pisano et al. [92]. The cat data would be in accordance with the early electrophysiological data presented by Angaut et al. [105] and Condé and Angaut [106], where no responses were mentioned in the main body of the RTN.

**Fig. 6:**
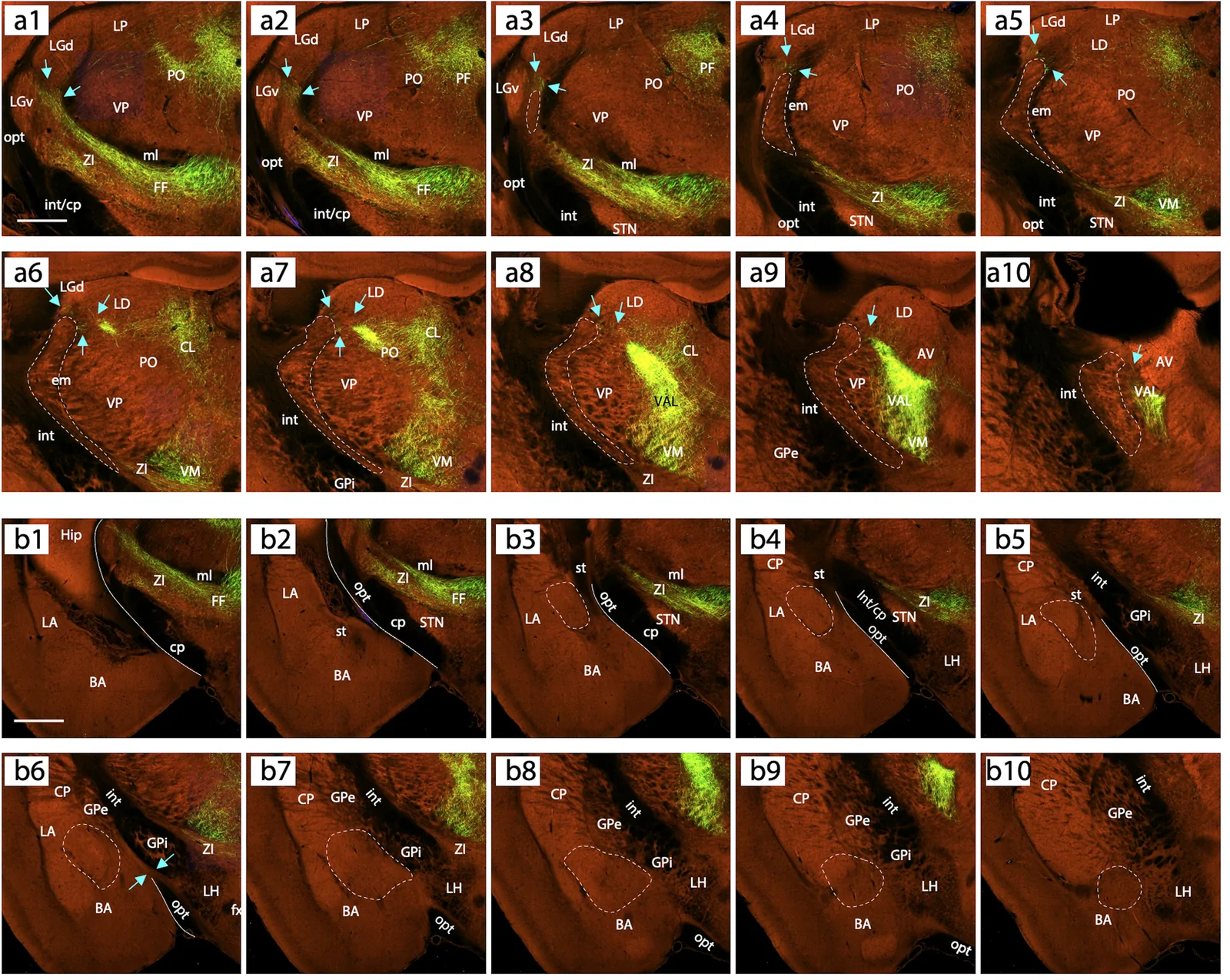
The lateral cerebellar nucleus does not provide projections to the reticular nucleus of the thalamus (RT) or the central nucleus of the amygdala (CEA). Material from experiment 268,389,532, made available by the Allen Brain Institute in the Mouse Brain Connectivity Atlas [26]. **a1-a10**: Two photon serial sections arranged consecutively from caudal (**a1**) to rostral (**a10**) showing labeled fibers and terminal arborizations resulting from the injection with AAV-EGFP into the lateral cerebellar nucleus shown in Fig. 5a. Note abundant labeling within the ZI continuing towards the LGv (**a1-2**). There, a small contingent of labeled fibers detaches just dorsal to the caudal tip of the RT (**a3**, RT is indicated by hatched contours, labeled fibers by cyan arrows). In **a4-10** these fibers (arrows) can be followed until they reach and terminate within the ventral LD, PO and VAL. No terminal arborizations, as would be indicated by small varicose fibers, are observed within the RT itself. **b1-b10**: Data from the same experiment but now focused on the amygdala. Sections are also arranged from caudal (**b1**) to rostral (**b10**), the contours of the CEA are indicated by a hatched line and incorporates its different subareas. The white line indicates the physical border between the diencephalon and telencephalon. Level **b6** shows the most-caudal passage between the lateral hypothalamus and amygdala (cyan arrows). However, note that neither here, nor rostral to this level, do labeled fibers use this passage to reach amygdala territory. Labeled fibers also do not enter the amygdala by way of the stria terminalis (st). Abbreviations: AV, anteroventral thalamic nucleus; BA, basal amygdala; CL, centrolateral thalamic nucleus; cp., cerebral peduncle; CP, caudate-putamen; em, external medullary lamina; FF, fields of Forel; fx, fornix; GPe, globus pallidus, external part; GPi, globus pallidus, internal part; Hip, hippocampus; int, internal capsule; LA, lateral amygdala; LD, laterodorsal thalamic nucleus; LGd, lateral geniculate nucleus, dorsal part; LGv, lateral geniculate nucleus, ventral part; LH, lateral hypothalamic area; LP, lateroposterior thalamic nucleus; ml, medial lemniscus; opt, optic tract; PF, parafascicular thalamic nucleus; PO, posterior thalamic nucleus; st, stria terminalis; STN, subthalamic nucleus; VAL, ventral anteriolateral thalamic complex; VM, ventral medial thalamic nucleus; VP, ventral posterior thalamic nucleus; ZI, zona incerta. Scale bars equal 500 μm

The study by Pisano et al. [92] describes several experiments to demonstrate the alleged connection between cerebellar nuclei and the RTN. Their initial observation was based on cerebellar cortical injections of the Herpes simplex virus strain H129-VC22, which was used as an anterograde transneuronal tracer. After survival times sufficient for only disynaptic transfer of the virus, anterograde label was found throughout large parts of the thalamus including the RTN. Herpes virus, however, is notorious for its degenerative aspects after infecting neurons [13, 15]. Hence, from the material (i.e. based on maximum intensity projections) shown in Pisano et al., it is difficult to decide if the labeling is within neurons, fibers or glia, while it also seems to take liberty with respect to the bounderies of the thalamic nuclei as, for instance, the width of the RTN in their Fig. 2C seems to be more than 500 micrometer, while, according to the atlas by Paxinos and Franklin [80], it would be only half that at its widest point. A subsequent, non-transneuronal, injection with AAV into the cerebellar nuclei allegedly confirmed the direct connections of the cerebellar nuclei with the RTN. However, their Fig. 3B basically only shows terminals in the ventral aspects of the RTN (caudal to level − 2.0), where its delineation with the LGNv and ZI is much more difficult to establish [80]. At levels rostral to −2.0, anterograde labeling is not convincing from the material shown. Subsequent experiments with injections of 200–300 nl of the retrogradely transported virus AAVrg in the RTN are hampered by the absolute requirement to keep the AAVrg within the limits of the RTN. In the supplemental material it is suggested that aberant labeling may have occurred by inadvertent viral uptake by passing fibers (i.e. labeling in the amygdala due to uptake by fibers of the stria terminalis) or leakage of the passing injection pipette (i.e. viral leakage in the hippocampus). This would suggest that also nuclear fibers coursing through the extertal medullary lamina that separates the ventral thalamic nuclei from the RTN to terminate more dorsorostrally within the VA/VL region could easily become infected (e.g. check experiments 304474221 and 268389532 of the Allen Brain Institute atlas on Mouse Connectivity Atlas) [26] (Fig. 6a). Hence, it would seem that presently claims as to a robust connection of the cerebellar nuclei with the RTN should be made with caution and are in dire need of replication before being allowed into the catalog of established cerebellar connections.

#### Is There a Direct Projection From the Cerebellar Nuclei to the Amygdala?

As a last example, we like to discuss recent evidence suggesting the existence of a direct connection from the cerebellar nuclei to the amygdala. Although it has been known that the cerebellum has an influence on the expression of fear and anxiety and short-latency responses have been recorded upon stimulation of the cerebellar nuclei using classic techniques [107, 108] the connection remained elusive to detect with anatomical techniques. Yet, recently, more modern techniques [109, 110], established direct connections between cerebellum and amygdala, which, in particular, concerns a direct monosynaptic connection from the lateral cerebellar nucleus to the central amygdala nucleus. According to the study by Cavdar et al., [109], this projection is mostly ipsilateral and reciprocated by projections from the amygdala to the lateral cerebellar nucleus, whereas Zhang et al. [110] claim a contralateral projection. A cerebellar nuclear connection to the amygdala has not been described before both in literature employing classic tracers [76, 77] as well as in several studies employing modern viral techniques [86, 111, 112], and could also not be discerned from data available at the Mouse Connectivity Atlas of the Allen Brain Institute [26] (Fig. 6b). Rather, studies suggest a disynaptic pathway involving the central and intralaminar thalamic nuclei as an intermediary between the cerebellar nuclei (in particular the fastigial nucleus) and the basolateral amygdala [112]. Yet, although direct nuclear projections to the amygdala could be seen in the supplemental material shown by Kebschull et al. [79], these were not explicitly mentioned and may have suffered from involving brainstem structures such as the parabrachial nuclei within the injection site (e.g. see their Figures S2 and S5) as these are a known afferent source of the amygdala [113–115] and also receive input from the central amygdala [116, 117]. Note, however, that this projection is bilateral though with an ipsilateral preponderance.

The study by Zhang et al. [110], conducted partly in rat, however, studied this connection using both anterograde, retrograde and transneuronal studies with viral tracers and found evidence that it is especially the dentate (lateral) nucleus that sends direct projections to the contralateral central nucleus of the amygdala. These studies seem to have been executed with great care and it is difficult to explain the difference with the mouse data that claim a disynaptic connection between the cerebellar nuclei and the amygdala with the intralaminar and central thalamus as intermediary [111, 112], as well as with earlier studies that did not report a direct cerebellar connection with the amygdala [76, 77]. Obviously, as the rat is a much larger animal than the mouse, spillage of the AAV2/9 virus resulting in primary infection within the parabrachial nuclei is less likely to have occurred. Also injections of AAV2/2-retro into the central amygdala would seem unlikely to have affected other direct target areas of the cerebellar nuclei, although it does not seem impossible that some injectate might have been spilled when the injection device passed the lateral thalamic border or ventral lateral geniculate nucleus [112, 118] and which, as a consequence, could have resulted in false positive labeling in the cerebellar nuclei [119, 120]. Also, as different strains of AAV behave differently with respect to their anterograde, retrograde and transneuronal tracing characteristics as studied in mice [3, 27, 60], it is also not inconceivable that the same virus strain may operate differently in different species (i.e. mice or rat). Finally, as some neurons in the parabrachial nuclei as well as in the hypothalamus have been described to provide both the cerebellum and the amygdala with collaterals [121, 122], one wonders if a combination of both retrograde and anterograde labeling within these neurons, followed by transneuronal transfer, could have resulted in the observed cerebello-amygdalar projections.

This possibility, however, seems unlikely as subsequent data provided by Zhang et al. [110] using fluorescent micro-optical sectioning tomography (fMOST) resulted in 3D-reconstructions of several single mouse dentate neurons together with their axon indeed supply the contralateral central amygdala. Moreover, the reconstructions show that these axons do not contain collaterals to other brainstem targets and reach the central amygdala following a virtually straight course. This would suggest that the amygdala-projecting neurons belong to an exclusive category as most excitatory projecting neurons of the cerebellar nuclei are generally recognized as a highly collateralizing population [66, 79]. Unfortunately, from the description, figure and video provided by the authors it is not quite clear how, i.e. by which route, the reconstructed axons reach the amygdala. Although the reconstructions suggest that the axon reaches the central amygdala from a caudomedial direction, the most caudal way for superior cerebellar peduncle fibers to enter the amygdala, however, would be by way of the ansa peduncularis, which would seem to require entry from a more medial to rostro-medial direction. Such a route, for instance, is taken by efferents from the parabrachial nuclei that reach the central amygdala (cf., note the course of fibers from the parabrachial nuclei in experiment 268415561 of the Mouse Connectivity Atlas [26]).

Finally, although the authors themselves state that the direct projection from the cerebellar nuclei to the central amygdala is minor, they still claim that this particular projection has a major impact on amygdalar functioning as not less than two-thirds of the investigated central amygdala neurons respond to selective optogenetic stimulation of only a part of this connection [110]. Hence, it seems remarkable that a connection that has not been described before in decades of investigation and by the authors themselves is described as minor can have such a profound influence on amygdalar functioning. However, as well as a cerebellar influence on reward centers as the VTA and SNc is sure to exist but, in our view, is more likely accomplished by way of multisynaptic pathways, it seems more probable that di- or polysynaptic pathways also will play a prominent role in a cerebellar effect on the control of anxiety [111, 112].

## Conclusion

It is without any reservation to state that viral tracers have brought on a whole new dimension to the study of both the anatomy and function of neuronal connections within the brain. As such they are and will be invaluable to the construction and understanding of functional neuronal networks. However, by the nature of the wide modifications, capabilities and characteristics of the used strains of virusses in their infection of different neuronal types, brain regions and species, scientists should be very careful in claiming important functional effect of connections, especially if these have not been described or generally accepted before using classical tracers. In particular, issues relating to the size of the effective injection site, possibility of infecting passing fibers, transneuronal passage, long incubation times, quality of transgenic mouse lines and section alignement with, and accuracy of, the used atlases, should be carefully evaluated. In the case of cerebellar impact on various processes, it would seem that the description of functional networks still needs additional work and may involve further integrating and processing steps not only in motor, but also in reward, cognition and anxiety functions.

## Data Availability

No new datasets were generated or analysed during the current study. Older datasets can be made available upon request.
